# Agenzia Italiana del Farmaco (AIFA): Developments and Strategy in a Transitioning European HTA Landscape

**DOI:** 10.3390/jmahp13010005

**Published:** 2025-02-05

**Authors:** Robert Nisticò

**Affiliations:** Agenzia Italiana del Farmaco, Via del Tritone 181, 00187 Rome, Italy; r.nistico@aifa.gov.it

## 1. Scope of AIFA’s Responsibilities and the Evolving Role of Health Technology Assessment

The Agenzia Italiana del Farmaco (AIFA) is the national public body that regulates medicines for human use in Italy. It governs pharmaceutical expenditure and follows the life cycle of a medicine to ensure its effectiveness, safety and appropriateness as well as access across Italy. AIFA operates independently, transparently and efficiently, under the guidance and supervision of the Italian Ministry of Health and under the supervision of the Ministry of Economy and Finance. A close collaboration has been established with all other stakeholders in the Italian Healthcare system, such as the Regions, the Instituto Superiore di Sanità, Scientific Research Institutes, Patient Associations, Clinical Societies, etc.

AIFA has a wide spectrum of responsibilities including the conduct and coordination of clinical research, authorization of innovative health technologies, management of health consumption and healthcare expenditure, promotion of health service search, surveillance and pharmacovigilance, conduct of audits and inspections and the management of health emergencies. Together with a few other agencies across Europe such as the Portuguese National Authority of Medicines and Health Products ‘INFARMED’ and the Norwegian Medical Products Agency ‘NOMA’, AIFA is in charge of both the national regulatory processes as well as pricing and reimbursement decisions.

Health Technology Assessment (HTA) is a key tool used in fulfilling AIFA’s responsibility. It aims to balance availability and access to innovative medicines, i.e., protecting and promoting public health by licensing safe and effective medicines on the one side and governing public pharmaceutical expenditure in order to guarantee sustainable and equal access to medicines on the other side. Complementary driving forces influence the time taken to achieve marketing authorization and patient access for new health technologies. Patient groups demand early access to potentially life-saving medicines and Health Technology Developers (HTDs) request favourable conditions for innovation and rapid access. On the contrary, payers, HTA organisations, and the clinical and scientific communities request strict safety assessments and thorough relative effectiveness data, which may delay respective timelines [1].

The role of HTA extends across the whole lifecycle of innovative medicines. Support for the evaluation of the benefit–risk ratio, analyses to guide pricing and reimbursement decisions and post-marketing authorization surveillance of appropriateness of utilization may all fall under the remit of HTA.

## 2. AIFA’s Reform: Structural Impact and Strategic Focus

AIFA is currently undergoing a reform that aims to promote investments in pharmaceutical research and development, to accelerate authorization processes, and to optimally prepare for upcoming challenges, e.g., considering the transitioning European environment.

**Figure 1:**
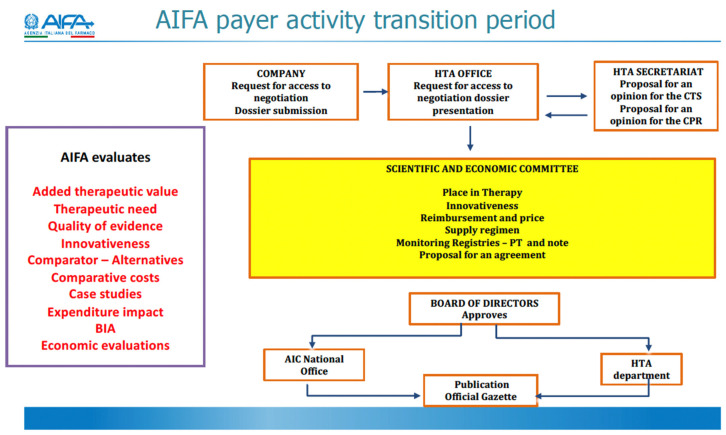
Overview AIFA payer activity transition period. (HTA: Health Technology Assessment; CTS: Technical Scientific Committee; CPR: Pricing and Reimbursement Committee; BIA: Budget Impact Analysis; PT: Pharmacotherapy.

Key elements of AIFA’s reform include the streamlining of approval processes, and the consolidation of the technical scientific commission (‘CTS’) and the pricing and reimbursement committee (‘CPR’) under one unique Scientific and Economic Committee. Key structural elements as well as the scope of AIFA’s activities are displayed in Figure 1. AIFA’s President is acting as a legal representative. Furthermore, one AIFA director each is responsible for the scientific and the administrative activities.

A key strategic focus area of AIFA’s current transition period is the advancement of early access policies for innovative cancer medicines. A recent scoping review and exploratory analysis addressed the clinical and economic impact of the current price and reimbursement negotiation scheme. Avoidable cases of cancer progression for selected cancer types occurring during the price negotiation process in Italy ranged from n = 404 (negotiation process for Pembrolizumab in MSI Colorectal Cancer) and n = 7102 (negotiation process for Abemaciclib in Breast Cancer). The total costs attributable to the progression of cancer during the negotiation process ranged from EUR 1.5 million for Atezolizumab and Bevacizumab in hepatocellular carcinoma to EUR 49.7 million for Abemaciclib in breast cancer [1]—indicating the need to accelerate the early access scheme in Italy. AIFA is actively involved in the respective activities of the European Medicines Agencies Network (EMAN) to facilitate the path to accessibility of new medicines across Europe.

## 3. AIFA’s Involvement in the EU HTA Process

The European Regulation (EU) 2021/2282 on Health Technology Assessment (EU HTAR) was adopted by the Council and the European Parliament in December 2021 and was effective as of January 2022 [2]. The regulation will be applied in a staggered manner starting from January 2025 for Joint Clinical Assessments (JCAs) of both cancer medicines and/or advanced therapy medicinal products (ATMPs), followed by orphan drugs from January 2028 and all other EU-centrally approved medicines from 2030 onward. Invasive or implantable, high-risk medical devices with CE marking may also be assessed via EU HTA, as of January 2026. The new European HTA Regulation introduces significant changes to the way Health Technology Assessments are conducted across Europe, with a goal to harmonize the evaluation of health technologies such as pharmaceuticals, medical devices, and (in vitro) diagnostic tools. Following is a breakdown of the EU HTAR and AIFA’s perspective, insights, and strategies in adapting to these developments.

AIFA supports the overall goal of the EU HTAR, which aims to improve the efficiency, consistency, and quality of HTA processes across the EU. From AIFA’s perspective, this harmonization is beneficial for ensuring faster access to new health technologies for Italian patients and avoiding redundant efforts in the evaluation of clinical evidence, allowing AIFA to focus on country-specific health needs such as pricing and reimbursement decisions. AIFA’s perspective remains firmly rooted in a patient-centered approach, ensuring that health technologies not only meet clinical standards but also improve patient outcomes and quality of life. The agency believes that the new regulation, by providing more structured and timely assessments, will help streamline the patient access process for critical treatments.

Despite the benefits of harmonization, AIFA emphasizes the importance of maintaining national autonomy in assessing non-clinical aspects of health technologies, such as cost-effectiveness, ethical considerations, and national healthcare priorities. The Agency views the ability to adapt HTA outcomes to the Italian healthcare context as crucial to ensuring that decisions reflect the needs of the Italian National Health Service (SSN).

AIFA further highlights the need for ensuring that country-specific factors, such as epidemiological trends and healthcare system structures, are adequately addressed during the national adoption phase.

Real-World Evidence (RWE) is recognized as having increasing importance in HTA processes and allowing for ongoing surveillance and re-assessment post-market. The Agency stresses the importance of using HTA as a mechanism to balance early access to innovation with the sustainability of the healthcare system and views HTA as a critical tool for determining which innovations deliver true value and merit investment.

## 4. Conclusions

AIFA is supportive of the EU HTAR. The Agency is determined to utilize the opportunity to take on the responsibility for both proactive involvement and system shaping on the European level and the possibility to streamline national decision-making across Italy. AIFA is in a process of increasing capacity and targeting competency profiles to actively prepare for the upcoming changes and challenges. Furthermore, it is intensifying collaboration with key stakeholders, such as other national HTA agencies across Europe, sharing knowledge and best practices to improve the overall efficiency and effectiveness of national and European HTA processes.
